# Psychological Studies on Hachisch and on Mental Derangement

**Published:** 1847-01

**Authors:** 


### Art. XV.

*Du Hachisch et de l'Aliénation Mentale, Etudes Psychologiques.* Par J. Moreau (de Tours), Médecin de l'Hospice de Bicêtre, Membre de la Société Orientale de Paris.—*Paris,* 1845.

*Psychological Studies on Hachisch and on Mental Derangement.* By J. Moreau (of Tours), Physician to the Hospital of the Bicêtre, and Member of the Oriental Society of Paris.—*Paris,* 1845. 8vo, pp. 431.

What is this Hachisch, and what it has to do with Mental Derangement, are questions which our readers will naturally ask, on perusing the above title; and to these questions it will answer our purpose better to give them a reply at once, than to play with their curiosity by keeping them in suspense, *Hachisch* is the oriental name of the plant which is scientifically known as the *cannabis indica,* and in our own vernacular as the Indian hemp; of which the extract has been lately used in this country as a sedative, narcotic, and antispasmodic. In the East, however, it is rather valued on account of its power of producing a species of intoxication, whose peculiar characters we shall presently analyse; and it is used for this purpose among the Arabs, as extensively as opium among the Turks and Chinese, and alcoholic liquors among the European nations. The most common preparation of Hachisch,—that which forms the foundation of all others, is a sort of fatty extract; this is prepared by boiling the leaves and the flowers of the plant with water to which a certain quantity of fresh butter has been added; the decoction is reduced by evaporation to the consistence of a syrup, and is then strained through a cloth. The butter thus becomes charged with the active principle, and acquires a greenish hue; its taste is very disagreeable, and hence it is seldom taken alone, but is mixed with other confections and aromatics, so as to form a sort of electuary. The one in most common use is termed by the Arabs *dawamesc;* but this is frequently mingled with substances of reputed aphrodisiac virtues, to enable it to minister more effectually to the grossly sensual purposes which seem to be the great object of life among many of the orientals. The leaves of the Indian hemp may be smoked with tobacco; and when freshly gathered they have a rapid and energetic action. Their peculiar powers are destroyed, however, by drying; whilst the dawamesc retains its activity for many years, the only change it undergoes being the acquirement of a slight rancidity, which does not seem to injure the vegetable extract, as M. Moreau informs us that he has had some of this substance in his possession for twelve years, and that it has lost none of its powers. We commend this fact to the attention of our pharmaceutists; for it certainly appears to us remarkable, that a substance of so little stability as to be altered by the simple drying of the leaves, should be preserved unaltered during so long a period by being mingled with fatty matter; and it suggests the question whether many of our more delicate and uncertain watery extracts might not be advantageously superseded by preparations made upon this plan.

There seems little doubt that the effects of the hachisch have been known from a very remote antiquity. The plant is very common in the centre and west of Asia, growing without any cultivation; and its peculiar properties, therefore, would be easily discovered. It is, indeed, remarkable how ingenious mankind have been in finding out means of inebriating themselves. The spontaneous fermentation of saccharine juices probably furnished the first intoxicating beverage; and we find in the oldest history of our race that the postdiluvian patriarchs were occasionally overcome with wine, in a manner which renders it probable that drunkenness was one of the vices of the antediluvian nations. At the present time we have, as is well known, an immense variety of these alcoholic beverages; some produced by the simple fermentation of expressed vegetable juices, whilst in others the art of distillation is employed for their concentration; some again being prepared from saccharine matters artificially produced, as in the process of malting; and one (as far as we know, a solitary instance) being obtained from an animal secretion,—the Tartars, who are cut off from all ordinary sources of alcoholic supply, managing to obtain a spirituous liquor which they term *koumiss* from the milk of their mares, which is well known to be peculiarly rich in saccharine matter whilst correspondingly poor in butter. Then again in the tropical portion of the Old World, we have the opium and the Indian hemp, and the tobacco in the New; whilst among some of the nations of Siberia, whose inhospitable climate will neither bring these productions to perfection, nor supply the means of obtaining alcoholic drinks, we find an extraordinary kind of intoxication induced by an agariciform fungus, the *amanita muscaria.*

The use of all these substances appears to have commenced at a period anterior to any historical or traditional record. We find numerous allusions to intoxicating draughts not only in sacred history, but in the oldest of the classic writers. Thus it will be remembered that Homer makes Helen give to Telemachus, in the house of Menelaus, a potion prepared from the *nepenthes,* which made him forget his misfortunes; and this plant was said to have been given to Helen by a woman of the Egyptian Thebes. We learn from Diodorus Siculus that on this circumstance much stress was laid by the Egyptians, who argued from various allusions in his writings that Homer had sojourned among them; for the women of Thebes, or as it was afterwards named Diospolis, were noted for possessing a secret by which they could dissipate anger or melancholy. The celebrated oriental scholar, M. Sylvestre de Sacy, appears to have made it pretty plain, that our word assassin is derived from the Arabic name of the Indian hemp. It is well known that it was originally employed in Syria to designate the followers of the "Old Man of the Mountain;" who were accustomed to devote themselves with blind obedience to the execution of his orders, however barbarous their character. These followers, according to the old traveller Marco Polo, were selected from the most robust young men of the country which was under this singular domination; their education tended in every way to impress upon them the duty of blind obedience, in return for which they were promised after death all the sensual pleasures they could imagine; and a foretaste of these was every now and then given to them by intoxicating them with hachisch, in the midst of scenes in which everything was provided to gratify their senses. In this manner a sort of fanaticism was gradually induced, which made the *Hachischins* (from a corruption of which name the word assassin was formed) ready to sacrifice either themselves or others at the direction of their chief, without the slightest hesitation. From the relations of various of the older travellers in the East, it appears that the peculiar intoxicating powers of this plant have long been made subservient to the purposes of the priestly classes, in practising upon the credulity of the vulgar; and for anything we know, the same use may be made of them at the present time. The employment of the extract of hemp is more extended than M. Moreau seems aware of. His own knowledge of it is confined to the East; but from the accounts of travellers, it would seem to be nearly as much used by the Hottentots (and probably by other African nations), and even by the aborigines of Brazil. Of the identity or difference between the American, African, Asiatic, and European species of hemp we are not able to speak positively; but there seems little doubt that they all possess the same active principle; the amount of it which is produced, however, varying with the nature of the climate in which the plant grows. Of the extract obtained from the common hemp (cannabis sativa) Endlicher says, "Emollitum exhilarat animum, impotentibus desideriis tristem stultam lætitiam provocat, et jucundissima somniorum conciliat phantasmata."

Whatever opinion we may hold in regard to the much-vexed question of the connexion between mind and body, there can be no doubt of the influence which the condition of the latter exerts over the operations of the former; and there are no more striking examples of such an influence, than those which are presented by the introduction of alcohol, opium, hachisch, nitrous oxide, or some other intoxicating substance into the current of the circulation. That the presence of a minute portion of any of these substances,—a portion almost too minute to be recognized by ordinary chemical processes,—in the blood which is passing through the capillaries of the brain, should so alter its relations to the nervous substance as to produce results which manifest themselves in an entire change of the ordinary course of psychical phenomena, must always be included, we apprehend, as a fundamental fact in any theory that may be framed by philosophers who please themselves with speculating on this mysterious question. The marked correspondence which may be traced between the phenomena of insanity and those which are induced by the introduction of such substances into the blood, must not be overlooked in any attempt to arrive at the true pathology of the former condition, or to bring it within the domain of the therapeutic art. We believe that Mr. Sheppard may claim the merit of having first prominently directed attention to this method of viewing the phenomena of insanity; and we would take this opportunity of stating our present feeling that in our unfavorable criticism of his little work 'Insanity a Blood-disease' (see Vol. XVII, p. 526), we had rather too strongly before our eyes the *demerits* of his hypothesis, than its positive value. His notion was, we are ready to admit, quite correct in regard to a certain class of cases of insanity: and his fault was that which is so common with young writers, namely, hasty generalization; the same idea being most unwarrantably stretched, so as to include *all* forms of this disease. There can be no doubt that the properties of the blood may be perverted by abnormal changes going on within the system, as well as by the direct introduction of poisonous substances from without; and its due relations to the nervous structure may be thus completely changed, so that the psychical operations are seriously interfered with, and a form of insanity develops itself, which is capable of being removed by the adoption of measures calculated to eliminate the morbific matter from the blood, and to restore it to its pristine purity. And we have little doubt that a part at least of the phenomena of those forms of insanity which are brought on by what are commonly termed *moral* causes, are referrible to the same agency; for every physiologist well knows how much the excitement of the passions and emotions involuntarily and indeed unconsciously affects those organic functions by which the blood is prepared and renovated; and how speedily any imperfection in the depurating actions (those of the liver and kidney more especially) is manifested in the abatement or irregularity of the functional powers of the nervous centres. We believe that an attentive study of the etiology and phenomena of insanity will gradually lead to the establishment of well-marked distinctions between this class of cases, and that in which disease of the cerebral structure itself is the proximate cause of the disordered psychical manifestations; and that in proportion as this difference is kept in view will be the clearness of our prognosis and the efficiency of our remedial measures.

We are rather surprised that this view of the subject has not occurred to M. Moreau, whose treatise is almost purely psychological, his principal object being to show that there is a positive *identity*—not merely the *analogy* usually admitted—between the psychical states of insanity and dreaming; the phenomena of the excitement produced by hachisch and other intoxicating agents, serving, in his opinion, to connect the two in such a manner as to prove that there is no essential difference between them. On this question we shall offer a few remarks as we proceed; stopping first to notice the principal effects of the *hachisch* upon the bodily system, and then endeavouring to analyse the chief psychical phenomena which result from its use.

The account of M. Moreau is principally founded upon his own experience; one characteristic of the condition which is thus induced being that the individual does not altogether lose his power of self-restraint and self-observation, and that he is subsequently able to retrace most of what he has felt and acted during the state of excitement. "I am satisfied," he remarks, "that by a certain exertion of the will, these effects may be altogether checked, or at least considerably diminished; just as we master the passion of anger by a strong voluntary effort." The effects vary considerably with the dose taken; but there is no certain rule for its administration, the results of the same dose being often very different in different individuals. There are some, indeed, on whom it seems to produce no effect whatever; no result having followed the administration of doses, which would have usually been followed by strongly marked phenomena. A small dose usually seems to have no other result than a moderate exhilaration of the spirits, or at most, to produce a tendency to unseasonable laughter; and this is generally one of the first effects of a dose which is large enough to occasion the "higher phenomena." There is at the same time a slight acceleration of the pulse with a somewhat retarded respiration; and a genial warmth diffuses itself through the body, with the exception of the feet which are usually chill. The wrists and forearms seem as if loaded with a weight; and movements are automatically performed as if to shake them free. At the same time vague muscular sensations, *des inquiétudes,* prompting to continual restlessness, are experienced in the lower extremities; corresponding, we presume, to what is known on this side of the channel as *the fidgets.* Other strange sensations, varying in different individuals, are very commonly experienced; many of them depending, we have no doubt, on the special direction of the attention to the part. Thus a very common feeling is that of the brain boiling over, and lifting the cranial arch like the lid of a tea-kettle. The epigastric region is very often the seat of similar odd feelings; the most common is a sense of anxiety or of constriction. The movements of the heart seem to the individual to be performed with unusual violence; but a bystander does not find that its impulse and sounds have undergone any increase, the change being in the sensorium of the subject of the experiment. Slight, or sometimes rather energetic spasmodic actions, never amounting however to proper convulsions, affect the limbs; the contraction of the flexor muscles predominating. There is usually a disposition to assume the recumbent position; and the limbs and trunk are then all brought together by their action. Sometimes the muscles of the face, especially those of the jaw, are affected with spasmodic twitches; and even a state of temporary trismus may supervene. These physical effects of the hachisch are usually but of short duration; but they often depart only to return again after an interval of variable duration. They commonly develop themselves most when the disturbance of the psychical functions has reached its height, or is taking its departure; and those who are in the habit of taking hachisch know how, by graduating the dose, to obtain the latter without the disagreeables of the former.

The first result of a dose of hachisch sufficient to produce the *fantasia* (as this remarkable condition is termed in the Levant), is usually an intense sentiment of *happiness,* which attends all the operations of the mind:

"It is really *happiness* which is produced by the hachisch; and by this I imply an enjoyment entirely moral, and by no means sensual as we might be induced to suppose. This is surely a very curious circumstance; and some remarkable inferences might be drawn from it, this for instance among others;—that every feeling of joy and gladness, even when the cause of it is exclusively moral, that those enjoyments which are least connected with material objects, the most spiritual, the most ideal, may be nothing else than sensations purely physical, developed in the interior of the system, as are those procured by the hachisch. At least, so far as relates to that of which we are internally conscious, there is no distinction to be made between these two orders of sensations, in spite of the diversity in the causes to which they are due; for the hachisch-eater is happy, not like the gourmand or the famished man when satisfying his appetite, or the voluptuary in the gratification of his amative desires, but like him who hears tidings which fill him with joy, like the miser counting his treasures, the gambler who is successful at play, or the ambitious man who is intoxicated with success," &c. (p. 54.)

We believe that our author is here quite correct in his general notion, although not very happy in his illustrations. Without having recourse to hachisch for the proof, we believe that most of our readers will be able to recall analogous states of exhilaration, and the reverse condition of depression in themselves; the former being characterized by a feeling of general well-being, a sentiment of pleasure in the use of all the bodily and mental powers, and a disposition to look with enjoyment upon the present, and with hope to the future; whilst in the latter state there is a feeling of general but indefinable discomfort, every exertion, whether mental or bodily, is felt as a burden, the present is wearisome, and the future is gloomy.* Such conditions are so distinctly referrible, in a great majority of cases, to the state of the general system, that we have no doubt whatever of their being universally dependent upon it. There are many individuals whose life is passed in an alternation between these two states, corresponding with alternations in the weather; the one being produced by a moderate north-easterly breeze and a bright sun, whilst the other is an invariable sequence upon a damp south-westerly wind and an over-clouded sky. In others, again, the depression is induced by a deficient biliary excretion; and the relief given by the depuration of the blood consequent upon a moderate dose of blue-pill is attended with positive exhilaration. Our readers will remember that we attempted in our last number (vol. XIV, p. 512) to establish the probability of the existence of a single seat of consciousness and of pleasurable or painful sensations, both for impressions received through the organs of the senses, and for the ideas formed by the instrumentality of the cerebrum. And it seems to us to correspond well with this view, that in the conditions to which we refer, the same feelings of pleasure and discomfort attend *all* the operations of the mind—the simple sensational, and the intellectual. In the state of exhilaration we feel a gratification from sensations which at other times pass unnoticed; whilst those which are usually pleasurable are remarkably enhanced; and in like manner, the trains of ideas which are started being generally attended with similar agreeable feelings, we are said to be under the influence of the pleasurable or elevating emotions. On the other hand, in the state of depression we feel an indescribable discomfort from the very sensations which before produced the liveliest gratification; and the very thoughts of the past, the present, and the future, which we before dwelt on with delight, now excite no feelings but those of pain, or at best of *insouciance*. There appears to us just as much reason for considering these simple feelings to be immediately dependent upon physical conditions of the central sensorium, as there is for regarding sensations themselves in that light; and in that case all our emotional states, however elevated their character may appear, are immediately dependent, as maintained by M. Moreau, upon such physical conditions; for, as we have attempted to show, there is no other essential difference between a lofty

* These, as all other moods of mind (we may almost say, as a matter of course), are delineated by Shakespeare. Thus, Romeo gives expression to the feelings inspired by the first state:

> My bosom's lord sits lightly in his throne,
> And, all this day, an unaccustomed spirit
> Lifts me above the ground with cheerful thoughts." (*Rom. and Jul.*, v, 1.)

While the reverse state is delineated by Hamlet, in his well-known soliloquy;

"I have of late—but wherefore I know not—lost all my mirth, forgone all custom of exercises; and, indeed, it goes so heavily with my disposition; that this goodly frame, the earth, seems to me a sterile promontory; this most excellent canopy, the air, look you,—this brave o'erhanging firmament, this majestic roof fretted with golden fire, why, it appears no other thing to me than a foul and pestilent congregation of vapours." (*Hamlet*, ii, 1.)

emotion or moral feling and a sensual gratification, than that the one consists of pleasure connected with certain *ideas,* whilst the other consists of pleasure excited by certain *sensations.* It is, then, in the elevation of the ideas which form a constituent part of the emotion, that the elevation of the sentiment really consists. As there are some individuals who by nature and habit attach themselves to sensual gratifications, so there are others who find their pleasure in the contemplation of the ideal; but these may really be as low in their moral nature as the preceding, for their ideas may be merely the recollections of past sensations of the most debased nature, as in the case of the enervated voluptuary who can no longer do anything but gloat over the past; and it is only when these ideas are habitually entertained, which have reference to loftier objects, that the real superiority of intellect over sense becomes apparent.

In the account which we have cited from M. Moreau, as to the peculiar sense of happiness resulting from the employment of hachisch, with the further light which we gain from other parts of his description, it appears to us that this peculiarity is to be discerned;—namely, that the feelings of pleasure are not excited so much by the *sensations* received from the external world, as from the *ideas* which are passing through the mind; and that where sensations *are* productive of pleasure, the pleasure is not sensational but ideal; in other words, the sensation itself does not give pleasure, but excites or modifies an idea which becomes a source of gratification. Now this is most remarkably seen in certain cases of dreaming. Every one knows how much the current of our thoughts in this condition is influenced by external impressions made upon the bodily system; of these impressions we are not conscious *as such;* but they arouse ideas, which become sources of pleasure or pain. Thus the application of a blister may cause a person to dream of being burned at the stake, and to feel the horrors of his ideal situation most acutely, whilst utterly unconscious of the real source of his torment; whilst, in like manner, a draught of cold air blowing upon us, may cause us to be transported in thought to the polar regions, and to suffer in imagination all the pains of a temperature of 40° below zero. We shall presently have occasion to notice the same class of phenomena as characteristic of insanity. Before proceeding with our account of the *fantasia,* we may stop to notice the remark of M. Moreau, that a peculiar state of general exhilaration or joyous excitement, closely analogous to that which is the first result of the hachisch, is often the precursor of an attack of insanity; showing, even in the mode of access of the disordered mental state, a close correspondence between the two conditions.

One of the first appreciable effects of the hachisch is the gradual weakening of that power of voluntarily controlling and directing the thoughts, which is so characteristic of the vigorous mind. The individual feels himself incapable of fixing his attention upon any subject; his thoughts being continually drawn off by a succession of ideas which force themselves (as it were) into his mind, without his being able in the least to trace their origin. These speedily occupy his attention, and present themselves in strange combinations, so as to produce the most fantastic and impossible creations. By a strong effort of the will, however, the original thread of the ideas may still be recovered; and the interlopers may be driven away, their remembrance however being preserved, like that of a dream recalling events long since past. These lucid intervals, however, become of shorter duration and can be less frequently procured by a voluntary effort; for the internal tempest becomes more violent, the torrents of disconnected ideas are so powerful as completely to arrest the attention, and the mind is gradually withdrawn altogether from the contemplation of external realities, being conscious only of its own internal workings. There is always preserved, however, a much greater amount of self-consciousness than exists in ordinary dreaming; the condition rather corresponding with that, in which the sleeper knows that he dreams, and if his dream be agreeable makes an effort to prolong it, and is conscious of a fear lest he should by awaking, cause the dissipation of the pleasant illusion. It is another characteristic of the action of the hachisch, that the succession of ideas has *at first* less of incoherence than in ordinary dreaming, and the ideal events do not so far depart from possible realities; and the disorder of the mind is at first manifested in errors of sense, in false convictions, or in the predominance of one or more extravagant ideas. These ideas and convictions are generally not altogether of an imaginary character, but are rather suggested by external impressions; these impressions being erroneously interpreted by the perceptive faculties, and giving origin, therefore, to fallacious notions of the objects which excited them. It is in the stage of the fantasia which immediately precedes the complete withdrawal of the mind from external things, and in which the self-consciousness and power of the will are weakened, that this perverted impressibility becomes most remarkable; more especially as the general excitement of the feelings causes the erroneous notions to have a powerful effect in arousing them:

"We become the sport of impressions of the most opposite kind; the course of our ideas may be broken by the slightest cause; we are turned, according to a common expression, by every wind. By a word or a gesture our thoughts may be successively directed to a multitude of different subjects, with a rapidity and a lucidity which are truly marvellous. The mind becomes possessed with a feeling of pride, corresponding with the exaltation of its faculties, of whose increase in energy and power it becomes conscious. It will be entirely dependent on the circumstances in which we are placed, the objects which strike the eyes, the words which fall on our ears, whether the most lively sentiments of gaiety or of sadness shall be produced, or passions of the most opposite character shall be excited, sometimes with extraordinary violence; for irritation shall rapidly pass into rage, dislike to hatred and desire of vengeance, and the calmest affection to the most transporting passion. Fear becomes terror, courage is developed into rashness, which nothing checks, and which seems not to be conscious of danger, and the most unfounded doubt or suspicion becomes a certainty. The mind has a tendency to exaggerate everything; and the slightest impulse carries it along. Those who make use of the hachisch in the East, when they wish to give themselves up to the intoxication of the *fantasia*, take great care to withdraw themselves from everything which could give to their delirium a tendency to melancholy, or excite in them anything else than feelings of pleasurable enjoyment. They profit by all the means which the dissolute manners of the East place at their disposal. It is in the midst of the harem, surrounded by their women, under the charm of music and of lascivious dances executed by the Almees, that they enjoy the intoxicating dawamesc; and with the aid of superstition, they find themselves almost transported to the scene of the numberless marvels which the Prophet has collected in his Paradise." (p. 67.)

The error of perception is remarkably shown in regard to time and space. Minutes seem hours, and hours are prolonged into years; and at last all idea of time seems obliterated, and the past and present are confounded together. This error seems to be easily accounted for, when we remember that our individual notions of time are founded upon the changes which occur in our own minds; so that a few minutes of very active succession of ideas may seem (as in a dream) like the lapse of years. But it does not appear so easy to account for the error in regard to space; except that every notion formed in this curious condition seems to partake of a certain exaggeration, and that the estimation of space in particular is greatly dependent upon that of time. M. Moreau mentions as an illustration, that on one evening he was traversing the passage of the Opera when under the influence of a moderate dose of hachisch. He had made but a few steps, when it seemed to him as if he had been there two or three hours; and as he advanced, the passage appeared interminable, its extremity receding as he pressed forwards. Here we think the error of time might suffice to explain the illusion. But he gives another more remarkable instance. In walking along the Boulevards, he has frequently seen persons and things at a certain distance presenting the same aspect as if he had viewed them through the large end of an opera-glass; that is, diminished in apparent size, and therefore suggesting the idea of increased distance. This erroneous perception of space is one of the effects of the *amanita muscaria,* or intoxicating fungus; a person under its influence being said to take a jump or a stride sufficient to clear the trunk of a tree, when he wishes only to step over a straw or a small stick. Such erroneous perceptions are common enough among lunatics, and become in them the foundation of fixed illusions; whilst in the person intoxicated by hachisch, there is still a certain consciousness of their deceptive character.

Though all the senses appear to be peculiarly impressible in this condition, yet that of hearing seems the one through which the greatest influence may be exerted upon the mind, especially through the medium of musical sounds. M. Theodore Gaultier, an artist of celebrity, describes himself as hearing sounds from colours, which produced undulations that were perfectly distinct to him. Here we recognize a little of the artistic imagination. But he goes on to say that the slightest deep sound produced the effect of the rolling thunder; his own voice seemed so tremendous to him, that he did not dare to speak out for fear of throwing down the walls, or of himself bursting like a bomb; more than five hundred clocks seemed to be striking the hour with a variety of tones, &c. &c. Of course those individuals who have a natural or an acquired musical ear, are the most likely to be influenced by the concord or succession of sweet sounds; and in such the simplest music of the commonest instrument, or even an air sung by a voice and in a style of the most mediocre kind, shall excite the strongest emotions of joy or melancholy, according as the air is cheerful or plaintive; the mental excitement being communicated to the body, and being accompanied with muscular movements of a semi-convulsive nature. This influence of music is not merely sensual, but depends like that of other external impressions, upon the associations which it excites, and upon the habitual disposition to connect with it the play of the imaginative faculties.

It is seldom that the excitement produced by the hachisch fixes itself upon any particular train of ideas, and gives rise to a settled delusion; for in general one set of ideas chases another so rapidly, that there is not time for either of them to engross the attention of the intellect, more especially since (as already remarked) there is usually such a degree of self-consciousness preserved throughout, as prevents the individual from entirely yielding himself up to the suggestions of his ideal faculties. M. Moreau mentions however that on one occasion, having taken an overdose and being sensible of unusual effects, he thought himself poisoned by the friend who had administered it, and persisted in this idea in spite of every proof to the contrary, until it gave way to another, namely, that he was dead and was about to be buried; his self-consciousness however being yet so far preserved that he believed his body only to be defunct, his soul having quitted it. But when this is altogether suspended, as it seems to be by a larger dose, the erroneous ideas become transformed into convictions, taking full possession of the mind; although sudden gleams of common sense occasionally burst through the mists of the imagination, and show the illusive nature of the pictures which they have conveyed to the sensorium. All this, as every one knows, who has made the phenomena of insanity his study, has its exact representation in the different stages of mental derangement; the illusive ideas and erroneous convictions being in the first instance capable of being dissipated by a strong effort of the will, gradually exerting more and more influence on the general current of thought, and at last acquiring such complete mastery over it, that the reasoning processes cannot be called into effectual operation.

Our readers must be well aware that there has been at different times much controversy among those who have attempted to investigate the mental pathology of insanity, in regard to the degree in which the feelings and affections may be deranged without intellectual disorder. Of late the general tendency of writers upon the subject has been towards the affirmative view of this question; admitting both a *general* disorder of the feelings, affections, and active powers, without any illusion or erroneous convictions impressed upon the understanding; and a *special* disorder caused by the exaggeration of some one particular feeling or propensity, which may exhibit its influence before any fixed delusion takes possession of the mind, and which may be regarded as the cause of that delusion where it exists. It is urged by M. Moreau, on the other hand, that although the feelings, affections, &c., may be in a state of excitement, either general or special, there is no abnormal character about the mental operations, until the intellect becomes so far disturbed that it cannot keep them any longer under restraint; and that, in consequence, there is no such thing as a purely moral insanity. The excited feelings have dominion over the mind, not so much on account of their own strength, as on account of the weakness of the reflective powers and of the will, which should keep them in check.—Such would certainly appear to be the case in the disordered mental condition induced by the hachisch; but although the phenomena may be to a certain extent the same as those of insanity, and may serve to elucidate them on many points, it seems to us that we must be careful of interpreting the latter altogether by the former, or supposing that the disorder always supervenes in the same way. It is reasonable to suppose that, when it is occasioned by the presence of morbific matter in the blood, or by any cause affecting the general circulation through the brain, all the powers and propensities of the mind should be more or less affected; and this we see in ordinary mania, the delirium of fever, puerperal mania, intoxication by alcohol, &c., as in the fantasia of hachisch or of opium. But it is still quite possible that one or more of the individual components of the healthy mental fabric should undergo alteration from some less general cause; whilst the remainder undergo no other change than that which results from the perverted action of the faculties or propensities which are primarily affected; and thus a monomaniacal state is produced, in which the individual is insane upon one point, whilst perfectly sane upon every other. It does not seem to us to be necessary, as M. Moreau thinks, that the intellectual powers should have undergone a considerable weakening, before the insane tendency or idea can acquire that predominance which shall allow it to operate upon the general current of thought, and to guide the determinations of the will. For anything which destroys the regular balance between the emotions and propensities on the one hand, and the will on the other, will cause the former to exert an undue influence: and this may happen, either by an extraordinary development of a particular propensity, the intellect and will retaining their normal power but being insufficient to keep it in check; or by a general weakening of the intellectual powers, which renders them unable to control even the ordinary propensities, and thus allows all of them full play or permits any one which is naturally strongest to assert its predominance; or lastly, both these changes may take place together, as seems to occur under the influence of hachisch.

It appears to us, however, that all the phenomena of disordered mental states will need to be analysed anew, in accordance with the progress of our knowledge of the physiology of the brain, and of the normal operations of the mind of which it is the instrument; and we shall now endeavour briefly to point out a few of the applications of which we believe the views we have propounded on that subject (in the article in our last Number to which we have already referred) to be capable, in aiding the elucidation of the numerous problems which these interesting conditions present for investigation. In the first place, the localization which we attempted to make of the *sensorium commune* (using that term in its limited sense, as the centre of consciousness,) appears to harmonize well with the fact, that its portals may be open to sensations immediately derived from the external world, whilst they may be closed to the perception of those cerebral operations, which, when we become conscious of them we call ideas: or that, on the other hand, they may be completely open to the latter, and altogether closed to the former. This last condition is that which presents itself when dreaming occurs in a sleep which is otherwise profound. The individual is altogether unconscious of external impressions, but he is vividly conscious of the operations of those parts of his encephalon, which were well designated by Reil as the "nerves of the internal senses." He does not always possess that peculiar sense of individuality or self-consciousness, which seems closely connected with the feeling of externality (if we may use the expression) that is called up by impressions made upon his organs of sense; for the ideas of which he is conscious, though relating to external things, seem not so strongly to call up the feeling of personal existence as do the sensations that first called them into being. When the self-consciousness or sentiment of individuality has a more distinct existence, as in reverie and some forms of dreaming, and also in somnambulism, it would seem as if the sensorium were less completely closed against impressions from without; although as we have already remarked, the attention is usually fixed so completely upon the cerebral sensations or ideas, that the influence of external impressions is not so much *direct* as *reflected* through them. This seems to us to be the explanation of the well known fact, that in many forms of somnambulism the individual seems to be conscious of those external impressions alone which either completely fall in with his train of thought, or which harmonize with it to a degree which renders them capable of directing and modifying it. And even in those other cases, in which the individual seems alive to every external impression, it seems as if he did not perceive them as ordinary sensations, but was conscious of them only through the ideas they excited.

In one instance of *induced* somnambulism also (by Mr. Braid's *Hypnotism*), a man remarkable for the poverty of his muscular development lifted with the greatest apparent ease a 28lb. weight *upon his little finger*, and swung it round his head, upon being assured that it was as light as a feather; whilst he declared himself altogether unable to raise a handkerchief from the table, after many apparently strenuous efforts, having been assured that its weight was too great for him to move. We have every reason to believe that the personal character of the individual placed him above the suspicion of intentional deceit; and if our readers will only try the first of these experiments for themselves in the waking state, we feel assured from our own experience that they will find great difficulty in accomplishing such a feat on the first trial, unless they should happen to possess a remarkable muscular development in their superior extremities; whilst the training necessary to enable it to be performed with the ease with which we saw it executed, ought to have manifested itself in the individual whose performance we witnessed, had that performance been the result of practice with a deceptive intention. Of course there is no *proof* of the absence of deception in the second experiment; and, therefore, we would not draw any inference from it alone. But both seem to agree in this remarkable phenomenon, which we think we may regard as *typical* of the state to which we allude; namely, that the actions of the individual were completely governed by his *ideas*, and that these ideas were not corrected by his *sensations* in the way that they are in the waking state. He was led to *believe* that the weight was light: and the *experience* of its actual pressure on his finger did not undeceive him. The degree in which our muscular sense and our muscular exertion are influenced by our mental condition, must be familiar to every one. Most of our readers have probably heard of Dr. Wollaston's exclamation, "Bless me, how heavy it is!" when he first poised a globule of potassium upon his finger, and judged of its weight from its metallic appearance; although it is really much lighter than water. And there is abundant evidence of the great increase of muscular power, which results from the confidence of success in any particular effort, or from the overpowering desire of saving one's life; whilst a doubt of failure, still more a settled determination of the impossibility of the success of the exertion, "unnerves" the frame, and deprives it of even its ordinary degree of power. We very well remember an experiment which was much in vogue a few years ago, in which four persons were to lift a fifth from the ground with extraordinary ease, provided that they all took a full inspiration at the same moment; and according to some accounts, the individual so elevated went up like a feather, and was capable of being sustained upon the fore-fingers of his bearers. Now it is easy to understand upon physiological principles the benefit of a full inspiration in communicating a temporary increase to the muscular power of those who were to exert it; but how it could make any difference in the weight of the party lifted whether his chest were full or empty (except by altering his specific gravity in an inappreciable degree) we never could understand; and after an attentive observation of the experiment, as performed under a variety of circumstances and by many different parties, we came to the conclusion, that the facility with which it was performed depended entirely (*cæteris paribus*) upon the strength of the impression previously made upon the mind of the parties; and that whilst those who were confident of the efficiency of the plan would accomplish the feat without effort, those who were sceptical could find out no other effect than that which might be predicted on the grounds already mentioned. If this explanation be fully carried out, and applied to cases in which the idea receives no contradiction nor correction from actual sensations, we believe that it will be found completely adequate to explain the remarkable phenomena we have mentioned. The whole character of the state of somnambulism, indeed, when carefully considered, appears to us explicable on this view,—that the mind is conscious only of ideas or cerebral operations, and that external impressions do not excite its attention as ordinary sensations, but only by exciting those operations, or in other words by generating ideas. We shall presently see that the phenomena of the *fantasia* and of insanity indicate that these states have many points of alliance with that which we have just been considering; but we shall first revert for a short time to the converse state, in which the sensorium remains open to external impressions, and the respondent consensual actions are performed; but in which it seems altogether incapable of being influenced by cerebral changes, that is, of receiving ideas or following out a process of reasoning.

Of this state, a remarkable case published not long since by a very intelligent observer,* affords a most apposite illustration. Our attention has been directed to it subsequently to the publication of the article to which we have already referred; and we could not wish a better practical exemplification of the theoretical views we then advanced. The subject of the case was a young woman of robust constitution and good health, who accidentally fell into a river and was nearly drowned. She remained insensible for six hours after the immersion; but recovered so far as to be able to give some account of the accident and of her subsequent feelings, though she continued far from well. Ten days subsequently, however, she was seized with a fit of complete stupor, which lasted four hours; at the end of that time she opened her eyes, but did not seem to recognize any of her friends around her; and she appeared to be utterly deprived of the senses of hearing, taste, and smell, as well as of the power of speech. Her mental faculties seemed to be entirely suspended; her only medium

* Mr. Robert Dunn, in Lancet, 1845.

of communication with the external world being through the senses of sight and touch, neither of which appeared to arouse *ideas* in her mind, though respondent *movements* of various kinds were excited through them.

"Her vision at short distances was quick; and so great was the sense of exaltation of the general sensibility upon the surface of the body, that the slightest touch would startle her; still, unless she was touched, or an object or a person was so placed that she could not help seeing the one or the other, she appeared to be quite lost to everything that was passing around her. She had no notion that she was at home, nor the least knowledge of anything about her; she did not even know her own mother, who attended upon her with the most unwearied assiduity and kindness. Her memory and the power of associating ideas [apparently the power of *forming* them also,—REV.] were quite gone. Wherever she was placed, there she remained during the day. She was very weak, but her bodily health was not much deranged; the tongue was clean; the skin moist; and the pulse quiet and regular. . . . . . Her appetite was good; but having neither taste nor smell, she ate alike indifferently whatever she was fed with, and took nauseous medicines as readily as delicious viands. She required to be fed. When I first saw her, she had no notion of taking the food that was placed before her; but, a few days afterwards, if a spoon was put into her hands, and filled by her mother, and conveyed a few times to her mouth, she would afterwards go on by herself until the whole was eaten. Her wants were sedulously attended to; but she *manifested no uneasiness as to food,* however long she might be kept without it. The same thing was observed in reference to drink. The calls of nature were alike unheeded by her; the urine and fæces were voided *unconsciously*; but with the striking peculiarity that, during the expulsion of the fæces, such was the reflex *action* induced, that the extremities became spasmodically convulsed and rigid, the head was thrown backwards, the muscles of the neck were stiff, and the eyelids closed; so that her mother considered that her bowels never acted without her having what she called "a convulsion fit;" the same thing occasionally happened when the bladder expelled its contents; and what is still more remarkable, the same tonic rigidity of the muscles invariably took place when she went to sleep."

Before quoting any more of the details of this interesting case, we may stop to remark, that all the automatic movements unconnected with sensation, of which the spinal cord is the instrument, seemed to go on without interference; as did also those dependent upon the sensations of sight and touch; whilst the functions of the other ganglia, together with those of the cerebral hemispheres, appeared to be in complete abeyance. The analysis of the facts stated regarding her ingestion of food seems to make this clear. She swallowed food when it was put into her mouth; this was a purely automatic action, the reception by the lips being probably excited by sensation (for this movement is different from that of suction, which has been shown to be purely reflex), whilst the act of deglutition, when the food is carried within reach of the pharyngeal muscles, is excited without the necessary concurrence of sensation. She made no spontaneous effort, however, to feed herself with the spoon; showing that she had not even that simple idea of helping herself, which infants so early acquire. But after her mother had conveyed the spoon a few times to her mouth, and had thus caused the muscular action to become associated with the sensorial stimulus, the patient continued the operation. It appears, however, to have been necessary to repeat this lesson on every occasion; showing the complete absence of memory for an idea of a character so simple and immediately connected with the supply of the bodily wants. The difference between an *instinct* and a *desire or propensity*, on which we formerly dwelt, is here most strikingly manifested. The patient had an instinctive tendency to ingest food; as is shown by her performance of the actions already alluded to: but these actions required the stimulus of the present sensation, and do not seem to have been connected with any notion of the character of the object *as food;* at any rate, there was no manifestation of the existence of any such notion or idea, causing her to manifest a *desire for food* or drink in the absence of the objects, when she must have been conscious of the uneasy sensations of hunger and thirst. The very limited nature of her faculties, and the *automatic* life she was leading, appear further evident from the following particulars.

"One of her first acts on recovering from the fit had been to busy herself in picking the bed-clothes; and as soon as she was able to sit up and be dressed, she continued the habit by incessantly picking some portion of her dress. She seemed to want an occupation for her fingers, and accordingly part of an old straw bonnet was given to her, which she pulled to pieces of great minuteness; she was afterwards bountifully supplied with roses; she picked off the leaves, and then tore them into the smallest particles imaginable. A few days subsequently, she began forming upon the table, out of these minute particles, rude figures of roses and other common garden flowers; she had never received any instructions in drawing.—Roses not being so plentiful in London, waste paper and a pair of scissors were put into her hands; and for some days she found an occupation in cutting the paper into shreds; after a time these cuttings assumed rude figures and shapes, and more particularly the shapes used in patchwork. At length she was supplied with proper materials for patchwork; and after some initiatory instruction, she took to her needle and in good earnest to this employment. She now laboured incessantly at patchwork from morning to night, and on Sundays and week-days, for she knew no difference of days; nor could she be made to comprehend the difference. She had no remembrance from day to day of what she had been doing on the previous day, and so every morning commenced *de novo.* Whatever she began, that she continued to work at while daylight lasted; manifesting no uneasiness for anything to eat or drink, taking not the slightest heed of anything which was going on around her, but intent only on her patchwork."

She gradually began, like a child, to register ideas and acquire experience. This was first shown in connexion with her manual occupation. From patchwork, after having exhausted all the materials within her reach, she was led to the higher art of worsted work, by which her attention was soon engrossed as constantly as it had before been by her humbler employment. She was delighted with the colours and the flowers upon the patterns that were brought to her, and seemed to derive special enjoyment from the harmony of colours; nor did she conceal her want of respect towards any specimen of work that was placed before her, but immediately threw it aside if the arrangement displeased her. She still had no recollection from day to day what she had done, and every morning began something new, unless her unfinished work was placed before her; and after imitating the patterns of others, she began devising some of her own. The first *ideas* derived from her former experience, that seemed to be awakened within her, were connected with two subjects which had naturally made a strong impression upon her; namely, her fall into the river, and a love-affair. It will be obvious that her pleasure in the symmetrical arrangement of patterns, the harmony of colours, &c. was at first simply *sensorial;* but she gradually took an interest in looking at pictures or prints, more especially of flowers, trees, and animals. When, however, she was shown a landscape in which there was a river, or the view of a troubled sea, she became intensely excited and violently agitated, and one of her fits of spasmodic rigidity and insensibility immediately followed. If the picture were removed before the paroxysm had subsided, she manifested no recollection of what had taken place; but so great was the feeling of dread or fright associated with water, that the mere sight of it in motion, its mere running from one vessel to another, made her shudder and tremble; and in the act of washing her hands they were merely placed in water An attempt to introduce the use of the shower-bath, by simply pouring water through a colander upon her head, induced such alarming excitement and fright, and was followed by a fit of insensibility of such long continuance, that the experiment was not repeated. We do not conceive that these phenomena warrant the inference that she had as yet any idea, even an indistinct one, of her accident, or of the circumstances attending upon it; for we have known a case of spontaneous double consciousness, in which a strong feeling excited in one state left an impression that manifested itself strongly in the other, although the individual was completely oblivious of its cause. But the manner in which the convulsions were brought on, in this case, shows that simple *ideas* were now being formed; for whilst the actual sight or contact of moving water excited them by the simple sensorial channel, the sight of a picture containing a river or water in motion could only do so by giving rise to the idea of water. Had she been able to comprehend the meaning of names in this state, we doubt not that the sight of the word *water* would have produced the same effect. Our readers will at once perceive the exact coincidence of these phenomena with those of the hydrophobic convulsion, to which we alluded on a former occasion; and will also agree with us, we trust, that the peculiar condition of this patient confirms the explanation of those actions which we then gave,—namely, that they are excited through the sensory ganglia; the *idea* transmitted from the cerebrum having exactly the same influence upon them as would have been exerted by a sensation directly transmitted from without.

From an early stage of her illness she had derived evident pleasure from the proximity of a young man, to whom she had been attached; he was evidently an object of interest when nothing else would rouse her; and nothing seemed to give her so much pleasure as his presence. He came regularly every evening to see her, and she as regularly looked for his coming. At a time when she did not remember from one hour to another what she was doing, she would look anxiously for the opening of the door about the time he was accustomed to pay her a visit; and if he came not, she was fidgety and fretful throughout the evening. When by her removal into the country she lost sight of him for some time, she became unhappy and irritable, manifested no pleasure in anything, and suffered very frequently from fits of spasmodic rigidity and insensibility. When, on the other hand, he remained constantly near her, she improved in bodily health, early associations were gradually awakened, and her intellectual powers and memory of words progressively returned. We here see very clearly, as it appears to us, the composite nature of the emotion of affection. At first, there was simple pleasure in the presence of her lover, excited by the gratification which former association had connected with the *sensation*. Afterwards, however, it was evident that the pleasure became connected with the *idea ;* she *thought* of him when absent, expected his return (even showing a power of measuring time when she had no memory for anything else), and manifested discomfort if he did not make his appearance. Here we see the true *emotion*, namely, the association of pleasure with the *idea ;* and the manner in which the *desire* would spring out of it. The desire, in her then condition, would be inoperative in causing voluntary movement for its gratification; simply because there was no intellect for it to act upon. Her mental powers, however, were gradually returning. She took greater heed of the objects by which she was surrounded; and on one occasion, seeing her mother in a state of excessive agitation and grief, she became excited herself, and in the emotional excitement of the moment suddenly ejaculated, with some hesitation, "What's the matter?" From this time she began to articulate a few words; but she neither called persons nor things by their right names. The pronoun "this" was her favorite word; and it was applied alike to every individual object, animate and inanimate. The first objects which she called by their right names were wild flowers, for which she had shown quite a passion when a child; and it is remarkable that her interest in these and her recollection of their names should have manifested itself at a time when she exhibited not the least recollection of the "old familiar friends and places" of her childhood. As her intellect gradually expanded, and her *ideas* became more numerous and definite, they manifested themselves chiefly in the form of *emotions ;* that is, the chief indications of them were through the signs of pleasure and pain. The last were frequently exhibited, in the attacks of insensibility and spasmodic rigidity, which came on at the slightest alarm. It is worth remarking that these attacks, throughout this remarkable period, were apt to recur three or four times a day, when her eyes had been long directed intently upon her work; which affords another proof how closely the emotional cause of them must have been akin to the influence of sensory impressions, the effects of the two being precisely the same. On one occasion, being alarmed by a stranger, she had quite an hysterical paroxysm, followed by insensibility; and in consequence she lost her speech, taste, and smell (which she had been gradually recovering) for some days afterwards. The mere sight of the same person again (evidently calling up the first disagreeable impression) was followed by a scream and excessive agitation.

The mode of recovery of this patient was quite as remarkable as anything in her history. Her health and bodily strength seemed completely re-established, her vocabulary was being extended, and her mental capacity was improving, when she became aware that her lover was paying attention to another woman. This idea immediately and very naturally excited the emotion of jealousy; which, if we analyse it, will appear to be nothing else than a painful *feeling* connected with the *idea* of the faithlessness of the object beloved. On one occasion this feeling was so strongly excited, that she fell down in a fit of insensibility, which resembled her first attack in duration and severity. This, however, proved sanatory.

"When the insensibility passed off, she was no longer spell-bound. The veil of oblivion was withdrawn; and, as if awaking from a sleep of twelve months' duration, she found herself surrounded by her grandfather, grandmother, and their familiar friends and acquaintances, in the old house at Shoreham. She awoke in the possession of her natural faculties and former knowledge; but without the slightest remembrance of anything which had taken place in the interval from the invasion of the first fit up to the present time. She spoke, but she heard not; she was still deaf, but as she could read and write as formerly, she was no longer cut off from communication with others."

From this time she rapidly improved, but for some time continued deaf. She soon perfectly understood by the motion of the lips what her mother said; they conversed with facility and quickness together, but she did not understand the language of the lips of a stranger. She was completely unaware of the change in her lover's affections which had taken place in her state of second consciousness; and a painful explanation was necessary. This, however, she bore very well, and has since recovered her previous bodily and mental health. We commend the attentive study of this interesting case (of which we have only given the leading features) to our readers, as illustrating the condition of a human being completely destitute as it would seem of cerebral power, and having only two sources of sensorial change, but these two in active operation, and serving as the direct stimuli to consensual movements, having associated with them also the simple feelings of pleasure and pain. As her power of forming ideas returned, pleasure and pain were associated with these ideas, constituting emotions; and as the higher powers of the intellect were not yet called into operation, these emotions were the dominant springs of her conduct, acting without the control of the reasoning processes, to which in the well-regulated mind they are subject, serving only as motives to them. Numerous cases might be alluded to, which present the same general features; but we know of none in which the details have been so carefully watched and recorded. Thus there is one mentioned by Dr. Rush, of a man who was so violently affected by some losses in trade that he was deprived almost instantly of all his mental faculties. He did not take any notice of anything, not even expressing a desire for food, but merely taking it when it was put into his mouth. A servant dressed him in the morning, and conducted him to a seat in his parlour, where he remained the whole day, with his body bent forward, and his eyes fixed on the floor. In this state he continued nearly five years, and then recovered completely and rather suddenly. There is also the well-known case of the sailor who was reduced to this condition by a fracture of the skull with depression; he remained in it for some time; and at last was immediately restored by the elevation of the depressed bone, which was effected by Mr. Cline. The Cretins of the first degree, also, are nearly in the same state. They spend their time in basking in the sun or in sitting by the fire (experiencing merely sensorial pleasure) without any traces of intelligence; and show no higher sensibility to the common wants of nature, than is evinced by their going when excited by hunger to places where they have been accustomed to receive their food. This, it will be observed, is a grade in the scale of intelligence somewhat above that exhibited in the cases already quoted. The condition of the Cretins of the second degree, who are more susceptible of education and can form simple ideas on the most common subjects, presents a remarkable parallelism with that of the young woman whose case we have been analysing, during the latter part of the time that preceded the second fit. And there is this further point of resemblance; that in this condition, the emotions and propensities, formed by the attaching of pleasure or pain to these ideas, are peculiarly strong, not being kept in check by any superior power.

We think it will be obvious to those who have followed us through our previous investigations, that phenomena of this kind afford very strong additional evidence in favour of our view of the functional distinctness of the sensory ganglia in man. The idiot mentioned by Dr. Rush, and the sailor on whom Mr. Cline operated, present all the psychical characters of M. Flourens' pigeon, from which the hemispheres had been removed. They were less alive to external objects, however, than the Cretins and Mr. Dunn's patient; in whom the sensory ganglia were evidently in a state of higher functional activity, and who obviously felt pleasure and pain in connexion with their sensations, these feelings manifesting themselves in the actions which they instinctively prompted. The formation of true emotions, as soon as ever ideas were generated with which feelings could be connected, is another point which seems to us to derive striking illustration from the phenomena we have adverted to. It is worth noticing in regard to the Cretins, that their various grades of idiocy correspond very closely to the successive stages of the development of the intellectual powers, which we find in the lower animals; and that the fact of the possession of full sensorial power with complete absence of intellectual, also harmonizes well with the fact that the sensory ganglia are fully developed, at a time when the cerebral hemispheres have scarcely made their appearance; so that their condition would seem to be one of pure arrest of development. We should not expect to find a complete absence of cerebrum in such cases; but we doubt not that an attentive examination would discover some imperfection, which suffices to prevent it from duly performing its functions.

We may seem to have wandered far from our original subject, in entering upon the preceding disquisition; but we have been desirous of laying the foundation for a new and more discriminating investigation of abnormal mental phenomena; and we have thought it best to commence with those cases in which the cerebral action is either suspended altogether, or is weak and inefficient, whilst the sensorial is unaffected. In most forms of imbecility, either congenital or acquired, this will be found, we believe, to be the general state. There does not seem to be an *error* of perception, so much as an *inability* to perceive, consequent upon deficiency of these powers of memory, comparison, &c., on which the formation of definite notions of external objects are dependent. This deficiency may not be complete, but may extend only to particular classes of objects; or it may not exist so far as external objects are concerned; being only manifested in the want of power to reason upon abstract ideas.

But there is also a certain class of imbeciles, in whom—to use the language of Hoffbauer—the *intensity* predominates over the *extensity;* that is, the mind is too much occupied with its own thoughts, and too little attentive to external objects. This is simply "absence of mind" carried to an excess, and constant instead of occasional; and the state appears to us to correspond closely with that of dreaming and some forms of somnambulism. The cerebrum is active, but feeds as it were upon its own thoughts; the sensory ganglia not being readily acted on by external impressions, nor easily communicating these to the intellect, which consequently remains isolated as it were from the external world, and cannot be brought *en rapport* with its condition.

We shall not enter at present into the analysis of the various forms of insanity, which result from perversion of the regular functions of the sensory ganglia and cerebrum, but shall confine ourselves to one point, on which M. Moreau dwells at great length, and which is in fact the chief "argument" of his book; namely, that in cases of monomania the delusion, where it exists, is purely intellectual, and is not induced by the exaltation of the particular moral feeling or emotion with which it seems to be connected. Our readers are doubtless aware that much discussion has taken place upon this question; the prevalent idea having been of late, that the disordered moral state is usually the cause of the illusion. The views we have propounded on the nature of the moral feelings tend to harmonize these conflicting doctrines, and to show that both are true in a certain sense. For if we consider a moral feeling to be (as its name almost implies) a simple feeling of pleasure or pain connected with a particular class of ideas, we see that where any such emotion exists in an exaggerated degree, it *must* involve an undue predominance of a certain class of ideas, which will manifest itself in the whole intellectual state of the individual, and in the interpretation he will put upon the occurrences going on around him. Thus, when we say that the depressing emotions (as is a very common occurrence in these days of eager competition with those who have overtasked their mental powers) are unduly predominant, we mean that painful ideas fill the mind; the individual looks at the past, present, and future in a gloomy light; he is thus led to give every possible unfavorable interpretation to the actions of others, even his dearest friends being in league to injure him; in a further stage of the malady, the patient's notions of actual occurrences are distorted by the erroneous perceptions which result from his habitual tendency to misinterpretation; and if the condition be still more exaggerated, he comes to believe in the reality, not merely of his view of the feelings of others towards him (judging of them by a reasoning process, in which the influence of his disordered feeling is sufficiently apparent); nor only of his erroneous interpretation of occurrences which have actually taken place, but of which the unpleasant character is entirely due to his habitual train of thought; but also of supposed occurrences, which have never had even the remotest foundation in fact, and which are altogether the products of his own disordered imagination. Thus we see that the tendency to indulge in a certain class of ideas, which is in itself purely intellectual, and the feeling which, when connected with those ideas, gives them their emotional character, are usually both concerned in the formation of hallucinations; and if our view is correct, it will be necessary to modify in a considerable degree the definitions now in vogue. As an illustration of how completely the true *propensities,* as well as the proper emotions, are dependent upon the formation of ideas, we may adduce an anecdote of a man who was strongly affected with the destructive tendency; and when restrained from doing mischief, he would manifest the pleasure he felt in dwelling on the idea, in the continual use of the words "crush," "smash," &c., which afforded a harmless vent to the passion that was struggling in his mind, at the same time that it indicated its nature. We now quit the subject for the present, to revert to it on some future opportunity.

